# Contrast-Associated Acute Kidney Injury in Patients with and without Diabetes Mellitus Undergoing Computed Tomography Angiography and Local Thrombolysis for Acute Lower Limb Ischemia

**DOI:** 10.1177/15385744211051503

**Published:** 2021-10-20

**Authors:** Talha Butt, Leena Lehti, Jan Apelqvist, Anders Gottsäter, Stefan Acosta

**Affiliations:** 1 Department of Clinical Sciences, 174435 Lund University, Malmo, Sweden; 2 Vascular Center, Department of Cardio-Thoracic Surgery and Vascular Diseases, Skåne University Hospital, Malmo, Sweden; 3 Department of Endocrinology, Skåne University Hospital, Malmo, Sweden

**Keywords:** acute lower limb ischemia, glomerular filtration rate, diabetes mellitus, computed tomography angiography, intra-arterial thrombolysis, contrast-associated acute kidney injury

## Abstract

Repetitive iodinated contrast media exposure may be harmful for the kidneys. The aim of the present study was to evaluate if patients with diabetes mellitus (DM) undergoing CT angiography (CTA) and local continuous thrombolysis for acute lower limb ischemia (ALI) had an increased risk of developing contrast-associated acute kidney injury (CA-AKI) compared to patients without DM. **Methods:** This is a retrospective study of patients undergoing CTA and local continuous thrombolysis due to ALI between 2001 and 2018. CA-AKI was defined as a 25% increase in serum creatinine within 72 hours after latest contrast administration. **Results:** There was no difference (*P* = .30) in the frequency of CA-AKI between patients with (27.9%; n = 43) and without DM (20.6%; n = 170). Among patients with CA-AKI, patients with DM had a lower (*P* < .001) estimated glomerular filtration rate (eGFR) at discharge (55 ml/min/1.73 m^2^) than at admission (70 ml/min/1.73 m^2^), while no such difference was found in the group without DM (*P* = .20). The gram-iodine dose/eGFR ratio was higher (*P* < .001) in patients with CA-AKI (median 1.49, [IQR 1.34-1.81]) than in those who did not develop CA-AKI (median 1.05 [IQR 1.00-1.13]). There was a trend that gram-iodine dose/eGFR ratio (OR 1.42/standard deviation increment, 95% CI 1.00-2.02; *P* = .050) was associated with an increased risk of CA-AKI, after adjusting for DM, age, and gender. **Conclusions:** The frequency of CA-AKI was high after CTA and local continuous thrombolysis for ALI without shown increased frequency for the DM group. Among patients with CA-AKI, however, patients with DM had worse renal function at discharge than those without DM. The gram-iodine dose/eGFR ratio in these patients needs to be lower to improve renal outcomes, particularly in patients with DM.

## Introduction

Acute lower limb ischemia (ALI) is a condition with a high risk of amputation and death^1^ and needs to be diagnosed and treated swiftly. The combination of a clinical examination and imaging is necessary to identify the location and extent of the arterial occlusion.^2^ Clinicians can choose between a number of different imaging modalities that can be used as a complement to clinical examination for diagnosing ALI. Computed tomography angiography (CTA) is the most commonly used modality in high-income countries due to its wide availability and high quality.^3^ Computed tomography angiography has also become a most important tool for planning of vascular procedures, in particular endovascular treatment.^4,5^

The risk of developing contrast-associated acute kidney injury (CA-AKI) is known to be higher in patients receiving not only a high amount of contrast medium but also repetitive contrast administration.^6^ Diabetes mellitus (DM) is commonly referred as a risk factor for CA-AKI but rather appears to amplify susceptibility in patients with underlying chronic kidney disease.^7^ The aim of the present study was to evaluate if patients with DM undergoing CT angiography and local continuous thrombolysis for ALI had an increased risk of developing CA-AKI compared to patients without DM.

## Patients and Methods

### Setting

The retrospective study took place in a tertiary referral vascular center with a primary care catchment area of approximately 750 000 and a regional catchment area of 1 650 000.

### Study Population

Between 2001 and 2018, 811 patients underwent leg revascularization due to ALI. A total of 213 patients, 43 with and 170 without DM, underwent CTA and local continuous thrombolysis with retrievable information regarding the amount of iodine contrast administered. There were 197 patients who had unilateral ALI and 16 patients who had bilateral (aorto-iliac occlusion) ALI. The median number of angiographies during the thrombolytic procedure was 2 (interquartile range [IQR] 2-3). According to the clinical memo, metformin was discontinued prior to examinations with iodinated contrast medium. This study was approved by the Swedish Ethical Review Authority (Dnr 2020-00 764).

### Thrombolysis

Before thrombolysis treatment is begun, several conditions must be met. Absolute contraindications are described in a local memo: operation or organ biopsy ≤ 2 weeks; cerebral infarction ≤ 6 weeks; cerebral metastasis, known arteriovenous cerebral malformations; and an epidural catheter or puncture of the dura ≤ 3 days. If none of these absolute contraindications are present several blood tests are analyzed, including creatinine, hemoglobin, aspartate aminotransferase, alanine aminotransferase, bilirubin, lactate dehydrogenase, activated partial thromboplastin time, prothrombin complex and platelet count. The patients are rehydrated with 1 L of Ringer’s acetate (Ringer’s acetate, Baxter, Kista, Sweden) if not on dialysis.

Preferably, a contralateral puncture is made in the common femoral artery; other access sites can be used if needed. Catheterization is made to the occlusion, and complementary high-dose thrombolysis (pulse spray), aspiration, and mechanical micro-fragmentation (AngioJet® device [MEDRAD, Warrendale, Pennsylvania, USA]; n = 42) are performed to shorten the treatment time. A flush catheter is placed in the occlusion, and the lytic agent alteplase, a recombinant tissue plasminogen activator, is deposited. Heparin bolus 5000 IE is given primarily, and during treatment, an infusion is given.

### Iodinated Contrast Media

Run-off CTA scanning was done from the hemidiaphragm to the forefoot. The iodinated contrast media used at CTA was usually Omnipaque™ (iohexol 350 mg I/ml, 90 ml, GE Healthcare Limited Little Chalfont, England). Contrast media injection from an antecubital vein was followed by 50 ml saline flush at a flow rate of 5 ml/s. The contrast media used at angiography were usually Omnipaque™ (iohexol 140 mg I/ml), Visipaque™ (iodixanol 270 mg I/ml, GE Healthcare Limited Little Chalfont, England), and Iomeron® (iomeprol 150 mg I/ml, Bracco Imaging Scandinavia AB, Gothenburg, Sweden).

Definitions The degree of ALI was classified according to the Rutherford classification.^8^ CA-AKI was considered if the patient had a 25% increase in serum creatinine within 72 hours after latest contrast administration.^9,10^ The estimated glomerular filtration rate (eGFR) was calculated with a simplified variant of the Modification of Diet in Renal Disease Study Group (MDRD) equation.^11^ DM was considered present if the patient had treatment with antidiabetic therapy with diet, oral hypoglycemic agents, or insulin. When the hemoglobin (Hb) level was below 134 g/L in men and 117 g/L in women, they were considered to have anemia. Patients were considered to suffer from ischemic heart disease (IHD) if they had a history of myocardial infarction, angina pectoris, coronary artery bypass, or percutaneous coronary angioplasty. Cerebrovascular accident (CVA) was defined as a history of stroke (cerebral bleeding or infarction) or transient ischemic attack. Only major amputations above the ankle were denoted as amputation. Preadmission and admission eGFR were collected at least > 90 days apart to distinguish habitual renal function from acute kidney injury, respectively.^12^

### Statistical Methods

All statistical analysis and data management were performed using SPSS for Windows, version 26.0 (IBM, Armonk, NY, USA). Continuous variables were expressed as median and IQR. The Mann–Whitney U test was performed for the comparison between continuous variables, and differences in proportions were analyzed with Pearson’s chi square test. Comparison of ordinal data was performed by Kendall’s tau-b test. Continuous variables such as age, and gram-iodine/eGFR ratio were tested for normal distribution with the Kolmogorov–Smirnov test, and these 2 variables were log-transformed due to skewed distribution. Odds ratios (OR) were expressed per 1 SD increment. Risk factors associated with CA-AKI were tested in a multivariable logistic regression analysis and expressed in terms of OR with 95% confidence intervals (CI). *P* values <.05 were considered significant.

## Results

### Patient Characteristics at Baseline

A total of 213 patients were included, of whom 43 had DM. One patient was on dialysis prior to thrombolysis. Patients with DM were older (*P* = .003), more often hypertensive (n = .002), more often had previous IHD (*P* = .005), CVA (*P* = .004), and lower GFR at admission (*P* = .046) (Table 1). The frequency of patients with infra-inguinal arterial occlusion was 48.8% (21/43) in patients with DM compared to 44.1% (75/170) in patients without DM (*P* = .58).

**Table 1.** General Characteristics in Patients with and without DM.

Factor	All patients, n = 213	Without DM, n = 170	With DM, n = 43	*P*-value
Median age (IQR)	70 (61.0-76.0)	69.0 (60.8-75.0)	73 (70.0-79.0)	.003
Female sex (%)	77 (36.2)	60 (35.5)	17 (39.5)	.60
Anemia (%)	45 (21.2)	36 (21.3)	9 (20.9)	.96
Hypertension	159 (74.6)	119 (70)	40 (93)	.002
Ischemic heart disease (%)	50 (23.5)	33 (19.4)	17 (39.5)	.005
^#^CVA (%)	30 (14.1)	18 (10.6)	12 (27.9)	.004
Median *eGFR (IQR), ml/min/1.73 m^2^ at admission	76 (57.0-93.0)	77.0 (57.0-95.3)	66.0 (51.0-86.0)	.046

^#^CVA = cerebrovascular accident; *eGFR = estimated Glomerular filtration rate. DM = diabetes mellitus. IQR = interquartile range

### Degree of Acute Lower Limb Ischemia at Baseline

There was no difference in degree of ALI between patients with vs without DM (Table 2).

**Table 2.** Degree of Acute Lower Limb Ischemia in Patients with and without DM.

Factor	All patients, n = 213 (%)	Without DM, n = 170 (%)	With DM, n = 43 (%)	*p*-value
Degree of ischemia—Rutherford class				
I	62 (29.1)	45 (26.5)	17 (39.5)	
IIa	90 (42.3)	75 (44.1)	15 (34.9)	
IIb	60 (28.2)	50 (29.4)	10 (23.3)	
III	1 (.5)	0 (.0)	1 (2.3)	.24

DM=diabetes mellitus.

### Iodine Contrast and Renal Function Outcomes

**Figure 1.**
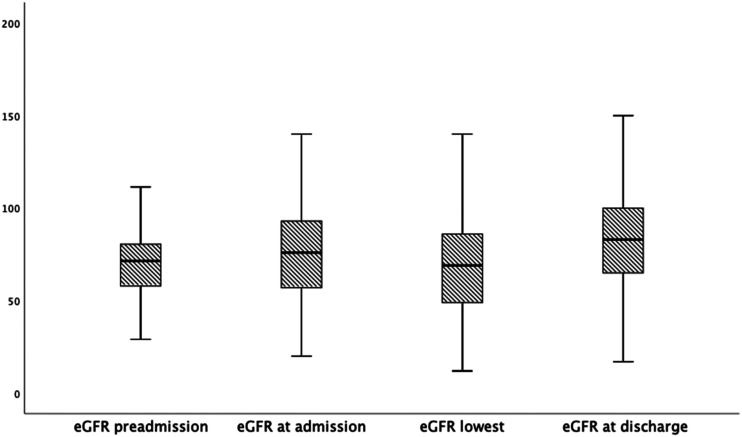
Estimated glomerular filtration rate at various time points from preadmission to discharge after thrombolysis for ALI in patients without DM (n = 170). Box plot graph showing median eGFR and IQRs. Lines across the boxes indicate the median, boxes represent IQR, and the whiskers are lines that extent from the box edge to the highest and lowest values, excluding outliers and extremes. Preadmission vs admission *P* < .001, Admission vs discharge *P* < .001. Admission vs lowest eGFR *P* < .001. Lowest eGFR vs eGFR discharge *P* < .001. Abbreviations: eGFR: estimated glomerular filtration rate; ALI: acute lower limb ischemia; DM: diabetes mellitus; IQR: interquartile range.

**Figure 2.**
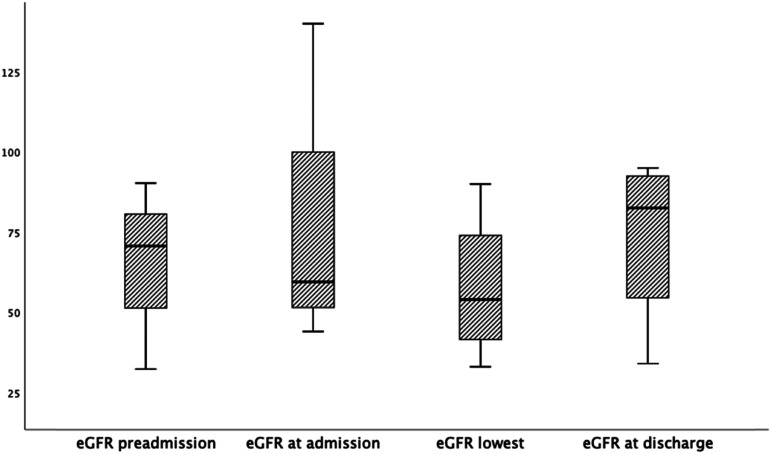
Estimated glomerular filtration rate at various time points from preadmission to discharge after thrombolysis for ALI in patients with DM (n = 43). Box plot graph showing median eGFR and IQRs. Lines across the boxes indicate the median, boxes represent IQR and the whiskers are lines that extent from the box edge to the highest and lowest values, excluding outliers and extremes. Preadmission vs admission *P* = .21, Admission vs discharge *P* = .22. Admission vs lowest eGFR P=.068. Lowest eGFR vs discharge *P* = .11. Abbreviations: eGFR: estimated glomerular filtration rate; ALI: acute lower limb ischemia; DM: diabetes mellitus; IQR: interquartile range.

There was no difference in the amount of iodine contrast media exposure at angiography between patients who were treated with adjunctive mechanical thrombolysis (n=42) or not (*P* = .95). Both the total amount of iodine contrast (*P* = .02) and the contrast amount given during the endovascular procedure (P=.037) were higher among patients without DM than those in the DM group. There was no difference in the frequency of CA-AKI between the groups (P=.30). Patients with DM had a lower eGFR at discharge (*P* = .018) (Table 3). The estimated glomerular filtration rate increased between preadmission and discharge after thrombolysis in both the group without DM (*P* < .001; shown in Figure 1) and in the DM group (*P* = .004; shown in Figure 2). The glomerular filtration rate increased between preadmission and admission in the group without DM (*P* < .001) but not in the DM group (*P* = .21). No patient required new onset of dialysis.

**Table 3.** Iodine Contrast Exposure and Renal Function Outcomes in Patients with and without DM.

Factor	All patients, n = 213	Without DM, n = 170	With DM, n = 43	*P*-value
Iodine contrast amount during CTA, grams (%)	31.5 (31.5-38.2)	31.5 (31.5-38.6)	31.5 (31.5-36.8)	.93
Median number of angiographies (IQR)	2 (2-3)	2 (2-3)	2 (2-3)	.73
Iodine contrast amount during thrombolytic procedure, grams (%)	25.6 (16.7-36.4)	26.8 (18.8-39.7)	19.2 (10.3-32.3)	.02
Total amount of iodine contrast, grams (%)	59.9 (48.0-76.0)	61.2 (48.6-77.0)	53.3 (44.4-67.6)	.037
Gram iodine/*eGFR≥ 1,0 (%)	73 (34.3)	58 (34.1)	15 (34.9)	.92
Contrast-associated acute kidney injury (%)	47 (22.1)	35 (20.6)	12 (27.9)	.30
Median *eGFR (^#^IQR), ml/min/1.73 m^2^ at discharge	82 (64.5-100.0)	84.5 (67.0-102-5)	70.0 (54.0-95.0)	.018

*eGFR=estimated glomerular filtration rate.

^#^Interquartile range.

DM=diabetes mellitus.

### Contrast-Associated Acute Kidney Injury

The gram-iodine dose/eGFR ratio was higher in patients with CA-AKI (median 1.49, [IQR 1.34-1.81]) than in those who did not develop CA-AKI (median 1.05 [IQR 1.00-1.13]), *P* < .001. Among patients with CA-AKI, there was no difference in median eGFR between admission (84 ml/min/1.73 m^2^) and discharge (77 ml/min/1.73 m^2^) in the group without DM (n = 35; *P* = .20), whereas the median eGFR decreased from 70 ml/min/1.73 m^2^ to 55 ml/min/1.73 m,^2^ respectively, in the DM group (n=12; P=.033). The total median amount of iodine contrast administered to patients with DM with CA-AKI was 52.6 g (IQR 44.6-60.1) compared to 58.5 g (IQR 50.8-81.0) for patients without DM and CA-AKI (*P* = .092).

When entering DM, the gram-iodine dose/eGFR ratio, age, and gender as covariates in a multivariable logistic regression model; there was a trend that the gram-iodine dose/eGFR ratio (OR 1.42/standard deviation [SD] increment, 95% CI 1.00-2.02; P=.050) was associated with an increased risk of CA-AKI (Table 4).

**Table 4.** Multivariable Analysis of Risk Factors Associated with Contrast-Associated Acute Kidney Injury after Thrombolysis for ALI.

Factor	OR (95% CI)	*P*-value
DM	.63 (.28-1.38)	.25
Age	1.06^a^ (.76-1.49)	.71
Male gender	1.21 (.61-2.40)	.59
Gram iodine dose/*eGFR ratio	1.42^a^ (1.00-2.02)	.050

*eGFR = estimated glomerular filtration rate.

^a^OR were expressed per 1 SD increment.

DM = diabetes mellitus.

ALI = acute lower limb ischemia.

### Non-Renal Outcomes

There was no difference in fasciotomy, major amputation or mortality at 30 days and 1 year between the 2 groups (Table 5). When entering age, DM, iodine/eGFR ratio, and Rutherford class 2b (motor deficit) as covariates and combined major amputation/mortality at 1 year as the dependent variable in a multivariable logistic regression analysis, no variable was found to be associated with major amputation/mortality at 1 year.

**Table 5.** Non-Renal Outcomes after Thrombolysis for Acute Lower Limb Ischemia in Patients with and without DM.

Factor	All patients, n = 213 (%)	Without DM, n = 170 (%)	With DM, n = 43 (%)	*P*-value
Fasciotomy	12 (5.6)	10 (5.9)	2 (4.7)	.75
Amputation 30 days	10 (4.7)	7 (4.1)	3 (7.0)	.43
Mortality 30 days	6 (2.8)	5 (2.9)	1 (2.3)	.83
Combined amputation/mortality 30 days	16 (7.5)	12 (7.1)	4 (9.3)	.62
Amputation 1 year	21 (9.9)	17 (10.0)	4 (9.3)	.89
Mortality 1 year	31 (14.6)	26 (15.3)	5 (11.6)	.54
Combined amputation/mortality 1 year	34 (16.0)	27 (15.9)	7 (16.3)	.95

DM = diabetes mellitus.

## Discussion

The present study of patients undergoing thrombolysis for ALI showed that renal function at discharge had improved, irrespective of the presence of DM or not. The repetitive contrast media exposure seemed to have contributed to a transient decline in renal function in patients without DM, whereas this could not be shown in DM. The rate of CA-AKI was 22%, similar to another high-risk cohort of patients.^13^ Compared to the 28% rate of CA-AKI among patients with DM in the present study, another recent study found a rate of 37% in patients with DM undergoing percutaneous coronary interventions (PCI) using the same definition of CA-AKI as in the present study, but the amount of iodinated contrast media administered was not stated.^14^

There might be several reasons for the lower doses of administered iodine contrast media in patients with DM than those without DM during thrombolysis. The occlusive lesions may be more distal in patients with DM, even if this could not be convincingly shown in the present study, necessitating less accurate angiographic imaging of the aorto-iliac segment and therefore less need of contrast media. In addition, vascular surgeons performing the thrombolytic procedures were aware of the calculated contrast volume threshold based on eGFR and knew whether the patient had DM or not, and therefore reduced iodine contrast load including dilution of contrast media when possible. Imaging with CO_2_ angiography^15^ to reduce iodine contrast exposure and spare renal function was also possible throughout the study period. It should be acknowledged that anatomic landmarks such as vascular calcifications, stents, and bony structures can all be used as visual aids during fluoroscopy. Modern equipment including road map, overlay, table position recall, and fusion imaging technology should be used to their full potential. Calculation of individual contrast thresholds^16^ and protocols for intravenous fluid expansions to prevent CA-AKI should be standard.^17^ For the sake of completeness, diagnostic imaging with ultrasound and open vascular surgery should not be forgotten as options in the management of patients with ALI to spare renal function.

The gram-iodine dose/eGFR ratio among patients developing CA-AKI was clearly higher than in those not developing CA-AKI in the present study, and there was a trend that the gram-iodine dose/eGFR ratio was associated with CA-AKI after adjusting for confounders. The iodinated contrast dose/eGFR ratio ≥ 1.0 used in the present study as a risk factor for developing CA-AKI has actually been reduced to < .5 in patients at risk of CA-AKI by the Swedish Society of Uroradiology (SSUR).^18^ In this context, the median gram-iodine dose/eGFR ratio of 1.05 in patients with ALI not developing CA-AKI was well above the new recommended threshold. Patients with ALI undergoing repetitive iodine contrast media exposure such as in the present study should all be considered high-risk patients for CA-AKI. Among patients with CA-AKI in the present study, a decrease in renal function at discharge compared to that at admission could only be shown in patients with DM, suggesting that these patients are more vulnerable to iodinated contrast media and at risk of overdosage of medications dependent of renal elimination. For instance, overdosage of metformin, an oral hypoglycemic agent with exclusive renal elimination, may result in lactic acidosis, and metformin should therefore be discontinued in all patients with DM and ALI before examination with repeated iodinated contrast medium.

Prognostic data are mainly derived from studies in patients undergoing PCI^19^ and might therefore not be completely applicable in patients undergoing lower extremity percutaneous peripheral arterial interventions. One difference is the presence of first-pass renal exposure of iodinated contrast media via the intra-arterial route in patients undergoing coronary intervention.^20^ CA-AKI was in the present study not shown to be associated with increased combined major amputation/mortality at 1 year, which is likely explained by the low sample size. Indeed, in a large study in 13 126 patients, it was possible to show that a creatinine clearance < 30 ml/min, high-contrast dose/GFR ratio, DM, and higher acuity of the peripheral vascular intervention procedure were independent predictors of CA-AKI. Second, CA-AKI was shown to be independently associated with in-hospital mortality, acute myocardial infarction, cerebrovascular accidents, vascular access complications, and blood transfusion.^21^

The retrospective and nonrandomized design of the present study were important limitations. Furthermore, the small study groups make comparisons prone to type 2 statistical errors. Adjustment for confounding is limited, and no adjustments for cardiovascular medication were taken into account in these emergently treated patients. The demonstrated increase in the eGFR between preadmission and discharge after thrombolysis both in patients with and without DM may likely be attributed to lower hydration levels before admission even if this time point was chosen to reflect the patients’ habitual renal function.^22^ Hence, in-hospital data on the eGFR at the preadmission time point, at least 90 days prior to admission, may have been collected when there could have been other reasons for dehydration. The strengths of the present study were the reliable information of amounts of iodine contrast medium given for calculation of the gram-iodine contrast dose/GFR ratio and the perioperative dynamic evaluation of renal function in patients with and without DM.

## Conclusion

The frequency of CA-AKI was high in the present cohort undergoing CTA and local continuous thrombolysis for ALI. Among patients with CA-AKI, patients with DM had worse renal function at discharge than those without DM. The gram-iodine dose/eGFR ratio in these patients needs to be lower to improve renal outcomes, especially in patients with DM. There are a number of ways as outlined to decrease iodine contrast dose exposure to reduce the risk of development of CA-AKI.
